# Supplementary material for: Status of the Archaeal and Bacterial Census: an Update
Source: mBio. 2016 May 17;7(3):e00201-16. doi: 10.1128/mBio.00201-16 (PMC4895100; doi:10.1128/mBio.00201-16)
Supplement: Table S1 — Description of environmental categories and the criteria used to assign sequences to each category. [file mbo003162817st1.pdf]

Supplemental Table 1. Description of environmental categories and the criteria used to assign sequences to each category.

Coarse	Fine	Description
Aerosol		Any sample taken from an atmosphere or aerosolized environment. Very rarely used.
Aquatic	Brackish	Any sample taken from a brackish or low-salt environment. Includes estuaries, brackish lakes, and other interfaces between salt and freshwater.
	Brackish sediment	A sample taken from soil or sediment layer of a body of water classified as Aquatic Brackish. Often used on brackish mud flats.
	Freshwater	Any freshwater sample. Includes rivers, ponds, aquifers, drinking water, and aquaculture habitats.
	Freshwater sediment	Any sample taken from the soil or sediment from any body of water that would be classified as Aquatic Freshwater.
	Marine	Any water sample from the ocean or any other saline aquatic source of water, including salt pans and salt lakes.
	Marine sediment	Any sample taken from the soil or sediment from any body of water that would be classified as Aquatic Marine.
	Hydrothermal vent	A sample taken from a hot spring or undersea vent, and any associated sediments.
	Ice	Icy samples. Includes glaciers, icebergs, and snowmelt.
	Other	A sample that eludes classification or had no specification.
Built	Digesters	A digester or other bioprocessing facility. Includes digesters, sewage treatment plants, microbial fuel production facilities, enrichment cultures that were found to degrade a certain compound, and septic tanks.
	Food-associated	A sample taken from human food or drink.
	Industrial/mining	A sample taken from an industrial process or a mine (active or abandoned). Includes factories, mines, and chemical plants.
	Pollution associated	Any sample found in an environment found with a pollutant or contaminant such as oil spills and environmental industrial waste. Much overlap with industrial.
	Other	A human built environment that eludes any above classification. Many clinical surfaces.
Plant associated	Plant root	Any sample taken from the root area of the plant. Includes rhizospheric soil and nodules.
	Plant surface	Any sample taken from the leaf, stem, or fruit surface of a plant. Used rarely because it usually isn't specified.
	Other	Any plant-associated sample that doesn't fit above classifications.
Soil	Agricultural soil	Any sample taken from farmland or garden soil. Sometimes overlapped with PR, but usually this category was used when the soil was not taken from rhizosphere.
	Desert soil	Any sample from arid or desert soil or sand. Includes desert rocks.
	Permafrost	Any sample taken from permafrost soil or from subglacial rock or soil.
	Other	Any sample that didn't fit above categories. Includes caves. Commonly used.
Host-associated	Vertebrate	Any vertebrate-associated sample. Body parts and animal fluids were often assumed to be human when not specified and thus put into this category.
	Arthropod	Any arthropod-associated sample. Includes larvae and feces.
	Other invertebrate	Any invertebrate animal that wasn't arthropod or vertebrate.
	Other	Any unspecified sample that could not be reasonably assumed to be human.
Other		Any sample that truly evaded all classification. The weirdest of the weird reside here.
