# Supplementary material for: Status of the Archaeal and Bacterial Census: an Update
Source: mBio. 2016 May 17;7(3):e00201-16. doi: 10.1128/mBio.00201-16 (PMC4895100; doi:10.1128/mBio.00201-16)
Supplement: Table S2 — Frequency that each bacterial phylum was sequenced before and after 2006. [file mbo003162817st2.pdf]

Supplementary Table 2. Frequency that each bacterial phylum was sequenced before and after 2006.

Phylum	<2006 and Before	After 2006	Total	Ratio of Ratios
Firmicutes	28567	448414	476981	1.17
Proteobacteria	34904	377724	412628	0.81
Actinobacteria	9524	210455	219979	1.65
Bacteroidetes	13869	135811	149680	0.73
Chloroflexi	1001	22337	23338	1.67
Cyanobacteria	1401	16781	18182	0.9
Acidobacteria	1168	14707	15875	0.94
Planctomycetes	822	14719	15541	1.34
Spirochaetae	1385	10406	11791	0.56
Fusobacteria	384	9074	9458	1.77
Verrucomicrobia	524	8084	8608	1.15
Tenericutes	1088	4618	5706	0.32
Lentisphaerae	117	3488	3605	2.23
Nitrospirae	324	2665	2989	0.62
Gemmatimonadetes	185	2725	2910	1.1
Saccharibacteria	120	2456	2576	1.53
Fibrobacteres	127	2413	2540	1.42
Atribacteria	38	2481	2519	4.89
Deinococcus-Thermus	357	1795	2152	0.38
Synergistetes	118	1709	1827	1.08
Aminicenantes	39	1692	1731	3.25
Deferribacteres	58	1490	1548	1.92
Chlorobi	155	1260	1415	0.61
Armatimonadetes	93	1319	1412	1.06
Marinimicrobia (SAR406 clade)	55	1260	1315	1.71
Parcubacteria	121	1169	1290	0.72
Kazan-3B-09	12	1224	1236	7.63
Gracilibacteria	33	1092	1125	2.48
Latescibacteria	47	950	997	1.51
Hydrogenedentes	33	922	955	2.09
Aquificae	592	321	913	0.04
Thermotogae	121	684	805	0.42
TM6	41	752	793	1.37
Microgenomates	141	617	758	0.33
Chlamydiae	169	552	721	0.24
Omnitrophica	16	617	633	2.89
TA06	28	546	574	1.46
OP3	36	536	572	1.11
Elusimicrobia	61	396	457	0.49
Cloacimonetes	135	307	442	0.17
Acetothermia	19	280	299	1.1
Aerophobetes	15	262	277	1.31
Caldiserica	23	229	252	0.75
Hyd24-12	8	234	242	2.19
WS6	36	199	235	0.41
SR1	4	186	190	3.48
SHA-109	7	168	175	1.8
PAUC34f	6	164	170	2.05
WD272	14	125	139	0.67
Thermodesulfobacteria	73	45	118	0.05
SM2F11	5	102	107	1.53
WCHB1-60	9	61	70	0.51
JL-ETNP-Z39	2	43	45	1.61
CKC4	0	41	41	NA
LCP-89	4	28	32	0.52
GOUTA4	5	26	31	0.39
Dictyoglomi	4	24	28	0.45
GAL08	4	13	17	0.24
SBYG-2791	0	16	16	NA
Chrysiogenetes	2	11	13	0.41
LD1-PA38	3	6	9	0.15
OC31	0	7	7	NA
RsaHF231	1	6	7	0.45
Calescamantes	2	1	3	0.04
S2R-29	0	2	2	NA
Total	98255	1312847	1411102	1.00
