# Supplementary material for: Status of the Archaeal and Bacterial Census: an Update
Source: mBio. 2016 May 17;7(3):e00201-16. doi: 10.1128/mBio.00201-16 (PMC4895100; doi:10.1128/mBio.00201-16)
Supplement: Table S3 — Frequency that each archaeal phylum was sequenced before and after 2006. [file mbo003162817st3.pdf]

Supplementary Table 3. Frequency that each archaeal phylum was sequenced before and after 2006.

Phylum	<2006 and Before	After 2006	Total	Ratio of Ratios
Euryarchaeota	3687	24719	28406	0.84
Thaumarchaeota	1032	16850	17882	2.04
Miscellaneous Crenarchaeotic Group	334	3404	3738	1.27
Crenarchaeota	537	643	1180	0.15
Woesearchaeota	114	504	618	0.55
Aenigmarchaeota	62	277	339	0.56
Aigarchaeota	79	223	302	0.35
Marine Hydrothermal Vent Group	7	260	267	4.63
Korarchaeota	39	175	214	0.56
Ancient Archaeal Group	0	191	191	NA
Nanoarchaeota	3	135	138	5.61
Miscellaneous Euryarchaeotic Group	7	78	85	1.39
Nanohaloarchaeota	2	77	79	4.8
Diapherotrites	16	23	39	0.18
SM1K20	4	29	33	0.9
Marine Hydrothermal Vent Group 1	4	9	13	0.28
Parvarchaeota	1	8	9	1
Marine Hydrothermal Vent Group 2	3	0	3	0
Unclassified	3	0	3	0
TVG8AR30	0	2	2	NA
Total	5934	47607	53541	1.00
