# Supplementary material for: Status of the Archaeal and Bacterial Census: an Update
Source: mBio. 2016 May 17;7(3):e00201-16. doi: 10.1128/mBio.00201-16 (PMC4895100; doi:10.1128/mBio.00201-16)
Supplement: Table S4 — Frequency that each bacterial phylum was found in each of the environmental categories. [file mbo003162817st4.pdf]

Supplementary Table 4. Frequency that each bacterial phylum was found across each of the environmental categories.

	Aerosol	Brackish	Brackish sediment	Freshwater	Freshwater sediment	Marine	Marine sediment	Hydrothermal vent	Ice	Aquatic other	Digesters	Food-associated	Industrial/mining	Pollution associated	Built other	Plant root	Plant surface	Plant other	Agricultural soil	Desert soil	Permafrost	Other soils	Vertebrate	Arthropod	Other invertebrate	Other host-associated	Other	Total
Firmicutes	939	88	69	1430	956	5000	2184	1459	184	1052	7886	7919	2556	3939	2144	3290	929	3399	2616	614	289	9302	363435	2148	678	2085	3179	476988
Proteobacteria	1921	705	210	11422	3405	53191	11209	5515	1151	4673	11076	3329	9115	18649	4293	11447	2668	3434	3765	570	698	21436	133674	6384	4209	7407	10038	412696
Actinobacteria	388	31	8	2815	454	3161	2894	172	257	619	842	240	904	6226	1110	2454	567	1016	923	701	447	10720	159964	940	492	269	1692	219985
Bacteroidetes	116	132	39	2328	445	21069	1943	684	365	752	4511	170	1062	4289	333	693	186	1157	343	188	110	3839	94839	1147	708	323	1596	149683
Chloroflexi	7	14	6	220	268	11894	1712	295	3	153	3504	11	386	894	124	165	60	97	187	283	21	1586	314	11	331	16	291	23340
Cyanobacteria	20	28	1	1098	28	9100	216	325	33	237	66	38	112	75	72	22	320	44	82	342	147	992	652	28	198	30	1126	18182
Acidobacteria	9	6	19	295	241	518	648	174	12	181	504	15	506	1215	97	839	61	134	1563	106	108	7079	454	63	317	8	308	15881
Planctomycetes	11	16	10	319	153	7516	1464	265	31	311	778	21	346	751	64	196	3	124	130	36	20	1163	496	95	154	81	302	15542
Spirochaetae	4	7	3	44	55	5803	246	42	1	55	403	3	111	129	18	2	0	3	1	8	0	71	1508	1183	30	10	85	11792
Fusobacteria	1	0	1	3	6	18	36	17	0	10	24	3	11	2	20	0	0	0	0	0	0	11	8494	3	1	440	15	9458
Verrucomicrobia	5	7	1	328	77	3337	107	99	17	93	95	0	117	397	35	200	2	88	66	37	28	731	2207	65	82	71	100	8608
Tenericutes	0	1	1	22	4	31	12	8	1	0	257	14	34	57	6	2	68	56	2	0	0	21	2373	94	57	58	17	5734
Lentisphaerae	0	7	3	47	42	2237	163	13	0	14	233	0	21	38	6	0	0	11	1	4	0	26	631	11	39	12	8	3605
Nitrospirae	10	5	4	262	91	77	291	96	0	237	121	23	275	183	58	98	0	13	55	10	7	671	61	4	43	2	108	2990
Gemmatimonadetes	1	2	8	42	43	245	176	20	2	15	29	2	102	695	21	123	0	28	178	71	12	738	149	3	30	0	71	2912
Saccharibacteria	4	3	0	23	7	18	4	4	6	22	80	3	43	374	6	29	13	15	11	8	3	182	1609	22	5	8	24	2576
Fibrobacteres	0	1	0	18	12	1429	30	2	0	8	42	2	3	8	2	1	0	3	3	1	0	17	199	721	12	0	7	2540
Atribacteria	0	0	0	0	28	78	2222	21	0	0	85	0	31	16	2	0	0	1	0	2	0	13	2	0	0	0	3	2519
Deinococcus-Thermus	14	1	1	61	12	216	16	227	4	30	34	5	162	86	30	8	5	13	11	33	1	198	519	3	2	2	89	2152
Synergistetes	1	0	0	6	13	16	4	10	0	1	895	2	95	44	5	0	0	10	1	0	0	18	560	59	0	1	10	1827
Aminicenantes	0	0	0	9	42	1193	337	22	0	3	30	0	9	17	12	1	0	0	3	1	0	38	0	0	7	0	1	1731
Deferribacteres	0	0	1	20	11	1058	74	31	0	8	28	0	39	20	1	0	0	1	0	1	0	20	185	5	1	0	1	1548
Chlorobi	7	21	1	186	57	285	75	88	1	36	155	0	85	73	38	14	1	11	13	1	19	63	28	16	12	0	49	1415
Armatimonadetes	3	2	0	36	21	424	72	38	2	17	107	4	41	74	5	23	9	30	20	9	7	224	179	2	1	1	23	1413
Marinimicrobia	0	1	0	1	1	1149	42	23	0	7	23	0	4	8	0	2	0	0	0	0	0	20	0	0	0	0	23	1315
Parcubacteria	0	5	0	118	36	328	207	83	1	31	151	0	48	70	16	11	0	9	14	0	1	76	50	0	3	0	22	1291
Kazan-3B-09	0	0	0	1	2	1181	47	1	0	2	0	0	0	1	0	0	0	0	0	0	0	1	0	0	0	0	0	1236
Gracilibacteria	4	2	1	34	19	744	52	96	0	18	15	0	11	11	4	1	0	4	0	0	0	10	57	9	5	4	11	1126
Latescibacteria	0	2	2	16	40	614	137	19	0	5	7	0	23	11	2	9	0	3	7	2	0	71	2	0	0	0	14	998
Hydrogenedentes	1	0	0	13	10	640	70	15	0	12	62	0	10	35	2	4	0	1	17	2	0	18	4	1	3	0	21	955
Aquificae	0	0	0	46	0	2	1	650	0	3	0	0	1	0	0	0	0	0	0	0	0	3	2	0	0	1	71	913
Thermotogae	0	0	0	7	8	39	9	89	0	2	368	0	106	30	2	2	0	7	1	1	0	21	0	0	0	0	9	805
TM6	1	1	0	27	11	327	114	7	1	19	36	2	29	34	2	13	0	19	2	0	1	75	19	0	3	9	27	793
Microgenomates	1	1	0	38	28	172	83	23	0	32	64	0	33	104	13	9	0	8	13	1	1	77	20	0	1	1	14	758
Chlamydiae	0	0	0	40	6	9	6	0	0	16	1	0	1	1	2	0	0	0	0	0	0	15	159	1	0	2	8	721
Omnitrophica	1	1	0	42	9	402	85	9	0	13	25	0	16	4	1	1	0	3	0	0	1	6	6	0	3	0	3	633
TA06	0	0	1	14	15	206	152	24	0	8	114	0	6	11	2	0	0	0	1	0	0	14	0	0	1	0	0	574
OP3	0	0	0	40	31	234	130	9	0	18	15	0	18	16	2	5	0	1	4	0	0	29	3	0	0	0	9	573
Elusimicrobia	0	1	0	28	2	9	15	18	0	13	9	0	18	25	2	7	0	4	9	1	1	57	47	179	0	0	5	457
Cloacimonetes	0	0	0	9	9	6	3	1	0	2	356	2	16	15	1	0	0	0	0	0	0	4	2	7	1	0	1	442
Acetothermia	0	2	0	56	7	24	148	18	0	4	3	0	4	3	1	0	0	0	0	5	0	4	0	0	0	0	8	299
Aerophobetes	0	0	0	20	0	4	234	2	0	1	0	2	1	1	0	0	0	0	0	0	0	5	0	0	0	0	2	277
Caldiserica	0	0	0	2	0	142	8	9	0	1	57	0	4	14	1	0	0	3	0	0	0	2	0	0	0	0	5	252
Hyd24-12	0	0	0	3	6	203	14	6	0	0	6	0	0	0	0	0	0	0	0	0	0	1	1	0	1	0	1	242
WS6	0	1	0	4	6	94	44	6	0	8	13	1	30	10	0	0	0	1	1	0	0	5	5	0	0	0	5	235
SR1	0	0	0	8	0	124	1	12	0	8	8	0	6	2	0	0	0	0	1	0	0	0	17	2	1	0	0	190
SHA-109	3	0	0	5	4	17	8	1	0	0	13	1	9	4	0	1	0	0	1	0	0	18	83	1	2	1	2	175
PAUC34f	0	0	0	2	0	45	10	3	0	0	1	0	2	0	0	0	0	0	0	1	0	2	7	0	42	0	2	170
WD272	0	0	0	2	2	0	0	0	1	5	1	0	3	7	0	4	0	0	0	3	0	108	1	0	0	0	1	140
Thermodesulfobacteria	0	0	0	8	0	0	1	85	0	0	0	0	4	1	0	0	0	0	0	0	0	1	0	0	0	0	0	118
SM2F11	0	0	0	9	0	62	6	1	0	0	3	0	0	0	0	15	0	0	1	0	0	3	5	0	0	0	1	107
WCHB1-60	0	0	0	3	2	1	1	0	0	0	6	0	2	16	1	2	0	1	4	0	0	22	5	0	0	0	2	70
JL-ETNP-Z39	0	0	0	4	3	12	10	0	0	0	0	0	0	4	0	0	0	0	1	0	0	9	0	0	1	0	1	45
CKC4	0	0	0	0	0	0	1	0	0	0	1	0	0	2	0	0	0	0	0	0	0	1	17	1	0	13	0	42
LCP-89	0	0	0	1	2	5	13	1	0	1	0	0	1	0	0	0	0	0	0	0	0	7	0	0	0	0	0	32
GOUTA4	0	0	0	7	3	1	2	1	0	1	2	0	2	1	0	2	0	1	0	0	0	6	0	0	0	0	1	31
Dictyoglomi	0	0	0	1	0	1	0	11	0	0	5	1	0	0	0	0	0	0	0	0	0	0	0	0	0	0	0	28
GAL08	0	0	0	2	0	0	0	9	0	0	0	0	4	0	0	0	0	0	0	0	0	2	0	0	0	0	0	17
SBYG-2791	0	0	0	0	0	16	0	0	0	0	0	0	0	0	0	0	0	0	0	0	0	0	0	0	0	0	0	16
Chrysiogenetes	0	0	0	0	0	0	3	0	0	0	1	0	2	0	0	0	0	0	0	0	0	2	0	0	0	0	0	13
LD1-PA38	0	0	0	0	0	0	8	0	0	0	0	0	0	0	0	0	0	0	0	0	0	1	0	0	0	0	0	9
OC31	0	0	0	0	0	0	1	0	0	1	1	0	2	1	0	0	0	0	0	0	0	0	0	0	0	0	1	7
RsaHF231	0	0	0	0	0	0	0	1	0	1	0	0	0	3	0	0	0	0	0	0	0	0	1	1	0	0	0	7
Calescamantes	0	0	0	1	0	0	0	0	0	1	0	0	0	0	0	0	0	0	0	0	0	0	0	0	0	0	0	3
S2R-29	0	0	0	1	0	0	0	0	0	0	0	0	0	0	0	0	0	0	0	0	0	0	0	0	0	0	1	2
Total	3472	1094	390	21647	6733	134727	27801	10860	2073	8760	33152	11813	16582	38696	8556	19695	4892	9753	10051	3042	1922	59855	773045	13209	7476	10855	19414	1411234
