# Supplementary material for: Status of the Archaeal and Bacterial Census: an Update
Source: mBio. 2016 May 17;7(3):e00201-16. doi: 10.1128/mBio.00201-16 (PMC4895100; doi:10.1128/mBio.00201-16)
Supplement: Table S5 — Frequency that each archaeal phylum was found in each of the environmental categories. [file mbo003162817st5.pdf]

Supplementary Table 5. Frequency that each archaeal phylum was found across each of the environmental categories.

	Aerosol	Brackish	Brackish sediment	Freshwater	Freshwater sediment	Marine	Marine sediment	Hydrothermal vent	Ice	Aquatic other	Digesters	Food-associated	Industrial/mining	Pollution associated	Built other	Plant root	Plant surface	Plant other	Agricultural soil	Desert soil	Permafrost	Other soils	Vertebrate	Arthropod	Other invertebrate	Other zoological	Other	Total
Euryarchaeota	2	488	138	598	726	1295	6	4099	5409	535	3688	117	1026	378	511	22	33	0	78	3	5261	1026	646	87	21	53	71	28410
Thaumarchaeota	0	687	274	546	280	805	36	6666	5505	146	843	0	156	36	173	0	108	0	149	35	126	752	116	0	42	0	129	17883
Miscellaneous Crenarchaeotic Group	0	92	107	101	224	357	0	102	2117	36	148	0	43	24	22	0	3	0	9	0	2	272	55	0	4	0	0	3738
Crenarchaeota	0	0	1	24	15	803	0	11	17	2	72	0	10	2	1	0	0	0	0	0	0	20	16	0	0	1	0	1180
Woesearchaeota	0	61	0	75	9	56	0	19	365	3	5	0	4	0	5	0	2	0	0	0	0	1	6	0	0	0	0	618
Aenigmarchaeota	0	8	2	24	59	70	0	21	100	25	1	0	0	0	0	0	0	0	1	0	0	0	20	0	0	0	0	339
Aigarchaeota	0	7	0	12	0	201	0	2	20	4	1	0	4	4	0	0	0	0	0	0	0	2	13	0	0	0	0	302
Marine Hydrothermal Vent Group	0	6	3	2	3	13	0	5	218	5	0	0	0	0	0	0	0	0	0	0	0	3	8	0	0	0	0	267
Korarchaeota	0	0	0	2	0	159	0	3	21	0	6	0	0	0	0	0	0	0	8	1	0	3	2	0	0	0	0	214
Ancient Archaeal Group	0	0	0	0	0	0	0	0	191	0	0	0	0	0	0	0	0	0	0	0	0	0	0	0	0	0	0	191
Nanoarchaeota	0	0	0	132	0	5	0	0	0	0	0	0	0	0	0	0	0	0	0	0	0	0	0	0	0	0	0	138
Miscellaneous Euryarchaeotic Group	0	0	0	6	4	10	0	1	61	0	0	0	0	0	1	0	0	0	0	0	0	1	0	0	0	0	0	85
Nanohaloarchaeota	0	5	0	11	0	0	0	48	2	12	0	0	0	0	0	0	0	0	0	0	0	0	0	0	0	0	0	79
Diapherotrites	0	13	0	1	2	4	0	3	6	1	0	0	0	0	0	0	0	0	0	0	0	7	0	0	0	0	0	39
SM1K20	0	1	0	6	2	6	0	0	14	3	0	0	0	0	0	0	0	0	0	0	0	0	0	0	0	0	0	33
Marine Hydrothermal Vent Group 1	0	0	0	0	0	10	0	2	0	0	0	0	0	0	0	0	0	0	0	0	0	0	0	0	0	0	0	13
Parvarchaeota	0	0	0	0	0	0	0	1	3	0	0	0	2	0	3	0	0	0	0	0	0	0	0	0	0	0	0	9
Marine Hydrothermal Vent Group 2	0	0	0	0	0	0	0	0	0	0	0	0	0	0	0	0	0	0	0	0	0	0	0	0	0	0	0	3
Unclassified	0	0	0	0	0	1	0	0	0	0	0	0	0	0	0	0	0	0	0	0	0	0	0	0	0	0	0	3
TVG8AR30	0	0	0	0	0	2	0	0	0	0	0	0	0	0	0	0	0	0	0	0	0	0	0	0	0	0	0	2
Total	2	1368	525	1540	1324	3797	42	10983	14049	772	4764	117	1245	444	716	22	146	0	245	39	5389	2087	882	87	67	54	200	53546
