# Supplementary material for: Status of the Archaeal and Bacterial Census: an Update
Source: mBio. 2016 May 17;7(3):e00201-16. doi: 10.1128/mBio.00201-16 (PMC4895100; doi:10.1128/mBio.00201-16)
Supplement: Table S6 — Frequency that each bacterial sequence or OTU was retrieved by cultivation or by culture-independent methods. [file mbo003162817st6.pdf]

Supplementary Table 6. Frequency that each bacterial sequence or OTU was retrieved by cultivation or by culture-independent methods.

Phylum	Sequences			OTUs		
	Cultured	Uncultured	Total	Cultured	Uncultured	Total
Proteobacteria	119742	292954	412696	9629	25152	34781
Firmicutes	84752	392236	476988	3073	29674	32747
Bacteroidetes	9744	139939	149683	2431	11146	13577
Actinobacteria	41122	178863	219985	2913	5222	8135
Planctomycetes	539	15003	15542	165	5134	5299
Acidobacteria	224	15657	15881	121	4571	4692
Chloroflexi	197	23143	23340	68	2964	3032
Verrucomicrobia	177	8431	8608	99	1396	1495
Cyanobacteria	3541	14641	18182	454	663	1117
Lentisphaerae	18	3587	3605	11	984	995
Fibrobacteres	62	2478	2540	34	784	818
Parcubacteria	2	1289	1291	2	676	678
Saccharibacteria	10	2566	2576	8	621	629
Armatimonadetes	10	1403	1413	6	545	551
Tenericutes	2182	3552	5734	210	275	485
Spirochaetae	2105	9687	11792	145	339	484
Microgenomates	0	758	758	0	468	468
Hydrogenedentes	5	950	955	2	441	443
TM6	5	788	793	3	436	439
Deinococcus-Thermus	554	1598	2152	122	310	432
Gemmatimonadetes	23	2889	2912	12	394	406
Latescibacteria	1	997	998	1	380	381
Gracilibacteria	3	1123	1126	3	376	379
Chlorobi	83	1332	1415	29	349	378
Fusobacteria	534	8924	9458	69	299	368
Marinimicrobia	8	1307	1315	3	322	325
OP3	0	573	573	0	321	321
Aminicenantes	0	1731	1731	0	319	319
TA06	0	574	574	0	287	287
Atribacteria	4	2515	2519	4	218	222
Nitrospirae	112	2878	2990	35	187	222
Deferribacteres	44	1504	1548	21	195	216
Kazan-3B-09	0	1236	1236	0	188	188
WS6	0	235	235	0	146	146
Elusimicrobia	4	453	457	3	143	146
Synergistetes	103	1724	1827	30	115	145
Cloacimonetes	2	440	442	1	134	135
Acetothermia	0	299	299	0	133	133
Caldiserica	4	248	252	2	120	122
SHA-109	0	175	175	0	110	110
Hyd24-12	0	242	242	0	103	103
Aerophobetes	1	276	277	1	96	97
Thermotogae	156	649	805	41	56	97
PAUC34f	1	169	170	1	94	95
Aquificae	211	702	913	50	43	93
SR1	0	190	190	0	83	83
Omnitrophica	0	633	633	0	83	83
Chlamydiae	536	185	721	52	29	81
WD272	0	140	140	0	73	73
SM2F11	0	107	107	0	72	72
WCHB1-60	0	70	70	0	58	58
JL-ETNP-Z39	0	45	45	0	37	37
Thermodesulfobacteria	21	97	118	8	28	36
GOUTA4	0	31	31	0	27	27
LCP-89	0	32	32	0	25	25
CKC4	2	40	42	2	18	20
Dictyoglomi	11	17	28	2	12	14
GAL08	0	17	17	0	11	11
LD1-PA38	0	9	9	0	8	8
OC31	0	7	7	0	6	6
SBYG-2791	0	16	16	0	6	6
RsaHF231	0	7	7	0	5	5
Chrysiogenetes	12	1	13	4	0	4
Calescamantes	0	3	3	0	3	3
S2R-29	0	2	2	0	2	2
Total	266867	1144367	1411234	19870	97515	117385
