# Supplementary material for: Status of the Archaeal and Bacterial Census: an Update
Source: mBio. 2016 May 17;7(3):e00201-16. doi: 10.1128/mBio.00201-16 (PMC4895100; doi:10.1128/mBio.00201-16)
Supplement: Table S7 — Frequency that each archaeal sequence or OTU was retrieved by cultivation or by culture-independent methods. [file mbo003162817st7.pdf]

**Supplementary Table 7. Frequency that each archaeal sequence or OTU was retrieved by cultivation or by culture-independent methods.**

| Phylum | Sequences | | | OTUs | | |
|---|---|---|---|---|---|---|
| | Cultured | Uncultured | Total | Cultured | Uncultured | Total |
| Euryarchaeota | 3352 | 25058 | 28410 | 522 | 2404 | 2926 |
| Thaumarchaeota | 46 | 17837 | 17883 | 25 | 896 | 921 |
| Miscellaneous Crenarchaeotic Group | 0 | 3738 | 3738 | 0 | 355 | 355 |
| Crenarchaeota | 215 | 965 | 1180 | 49 | 26 | 75 |
| Woesearchaeota | 0 | 618 | 618 | 0 | 70 | 70 |
| Aenigmarchaeota | 0 | 339 | 339 | 0 | 51 | 51 |
| Korarchaeota | 2 | 212 | 214 | 2 | 35 | 37 |
| Aigarchaeota | 0 | 302 | 302 | 0 | 31 | 31 |
| Nanoarchaeota | 1 | 137 | 138 | 1 | 24 | 25 |
| Marine Hydrothermal Vent Group | 0 | 267 | 267 | 0 | 22 | 22 |
| Ancient Archaeal Group | 0 | 191 | 191 | 0 | 15 | 15 |
| Nanohaloarchaeota | 2 | 77 | 79 | 2 | 8 | 10 |
| Diapherotrites | 0 | 39 | 39 | 0 | 8 | 8 |
| Miscellaneous Euryarchaeotic Group | 0 | 85 | 85 | 0 | 8 | 8 |
| SM1K20 | 0 | 33 | 33 | 0 | 7 | 7 |
| Marine Hydrothermal Vent Group 1 | 0 | 13 | 13 | 0 | 5 | 5 |
| Marine Hydrothermal Vent Group 2 | 0 | 3 | 3 | 0 | 2 | 2 |
| Parvarchaeota | 0 | 9 | 9 | 0 | 2 | 2 |
| TVG8AR30 | 0 | 2 | 2 | 0 | 2 | 2 |
| Unclassified | 0 | 3 | 3 | 0 | 2 | 2 |
| Total | 3618 | 49928 | 53546 | 601 | 3973 | 4574 |
